# Effect of neutrophil elastase and its inhibitor EPI-hNE4 on transepithelial sodium transport across normal and cystic fibrosis human nasal epithelial cells

**DOI:** 10.1186/1465-9921-11-141

**Published:** 2010-10-08

**Authors:** Virginie Prulière-Escabasse, Christine Clerici, Grégoire Vuagniaux, Andre Coste, Estelle Escudier, Carole Planès

**Affiliations:** 1 INSERM, U 955, Créteil, F-94000, France; 2 Université Paris Est, Créteil F-94000, France; 3 AP-HP, Hôpital Intercommunal et Groupe Hospitalier Henri-Mondor-Albert-Chenevier, Service d'Oto-Rhino-Laryngologie et de Chirurgie Cervico-Faciale, Créteil F-94000, France; 4 INSERM, U 773, CRB3, Paris F-75018, France; 5 Université Denis Diderot-Paris 7, F-75013 Paris, France; 6 AP-HP, Hôpital Bichat-Claude Bernard, Service de Physiologie, Paris, F-75018, France; 7 Debiopharm SA, Lausanne CH-1005, Switzerland; 8 INSERM, U 933, Paris F-75012, France; 9 Université Pierre et Marie Curie Paris 6 and AP-HP, Hôpital Armand Trousseau, F-75012 Paris, France; 10 Equipe d'Accueil EA 2363, Université Paris 13, Bobigny F-93009, France; 11 AP-HP, Hôpital Avicenne, Service de Physiologie, Bobigny F-93009, France

## Abstract

**Background:**

Hyperactivity of the epithelial sodium (Na^+^) channel (ENaC) and increased Na^+ ^absorption by airway epithelial cells leading to airway surface liquid dehydration and impaired mucociliary clearance are thought to play an important role in the pathogenesis of cystic fibrosis (CF) pulmonary disease. In airway epithelial cells, ENaC is constitutively activated by endogenous trypsin-like serine proteases such as Channel-Activating Proteases (CAPs). It was recently reported that ENaC activity could also be stimulated by apical treatment with human neutrophil elastase (hNE) in a human airway epithelial cell line, suggesting that hNE inhibition could represent a novel therapeutic approach for CF lung disease. However, whether hNE can also activate Na^+ ^reabsorption in primary human nasal epithelial cells (HNEC) from control or CF patients is currently unknown.

**Methods:**

We evaluated by short-circuit current (*I*_sc_) measurements the effects of hNE and EPI-hNE4, a specific hNE inhibitor, on ENaC activity in primary cultures of HNEC obtained from control (9) and CF (4) patients.

**Results:**

Neither hNE nor EPI-hNE4 treatments did modify *I*_sc _in control and CF HNEC. Incubation with aprotinin, a Kunitz-type serine protease inhibitor that blocks the activity of endogenous CAPs, decreased *I*_sc _by 27.6% and 54% in control and CF HNEC, respectively. In control and CF HNEC pretreated with aprotinin, hNE did significantly stimulate *I*_sc_, an effect which was blocked by EPI-hNE4.

**Conclusions:**

These results indicate that hNE does activate ENaC and transepithelial Na^+ ^transport in both normal and CF HNEC, on condition that the activity of endogenous CAPs is first inhibited. The potent inhibitory effect of EPI-hNE4 on hNE-mediated ENaC activation observed in our experiments highlights that the use of EPI-hNE4 could be of interest to reduce ENaC hyperactivity in CF airways.

## Introduction

Abnormalities in cyclic AMP-dependent chloride secretion and excessive sodium (Na^+^) reuptake by airway epithelial cells related to cystic fibrosis transmembrane conductance regulator (CFTR) deficiency are thought to alter fluid homeostasis at the airway surface liquid leading to dehydration, impaired mucociliary clearance, and infection [1]. Activation of CFTR Cl^- ^channel is known to inhibit epithelial Na^+ ^channel (ENaC) in normal native airway epithelial cells. In CF airways, mutation of CFTR leads to increased ENaC activity with increased transepithelial Na^+ ^and water reabsorption [2-5]. Indeed, it has been shown that overexpression of the β-ENaC subunit in mouse airways increases Na^+ ^reabsorption, decreases mucociliary and bacterial clearance and leads to airway inflammation and obstruction, and to a cystic fibrosis-like disease [6]. Therefore, inhibition of ENaC activity in the airways has been proposed for treatment of CF pulmonary disease.

Despite its physiological importance in lung fluid homeostasis, the tissue-specific regulation of ENaC in airways is still poorly understood. Most studies have focused on the systemic regulation of ENaC by hormones [7], but the role of extracellular luminal factors present in the immediate vicinity of the channel has been scarcely investigated. In recent years, the concept of an autocrine regulation of ENaC by epithelium derived extracellular serine proteases has emerged from several observations [8,9]. In 1997, using functional complementation assays to detect increases in ENaC activity in the *Xenopus *kidney A6 renal cell line, Vallet *et al *(10) cloned a trypsin-like serine protease, the channel-activating protease 1 (CAP1). This glycosylphophatidylinositol-anchored protease increased amiloride-sensitive Na^+ ^current when coexpessed ENaC in *Xenopus *oocytes [10,11]. ENaC activation was fully prevented by extracellular addition of the serine protease inhibitor aprotinin and mimicked by external tryspsin. Mammalian homologs of *Xenopus *CAP1, such as mouse mCAP1 or human and rat prostasin, were also shown to activate ENaC in the *Xenopus *oocytes expression system [12-15]. More recently, additional transmembrane serine proteases activating ENaC have been identified in mammals, including channel-activating protease 2 (CAP2) and channel-activating protease 3 (CAP3) cloned from the mpkCCD_d4 _mouse kidney cell line [14], TMPRSS3 from human inner ear [16], or TMSP-1 from rat kidney [17]. The precise mechanism for protease-mediated activation of ENaC has not been fully elucidated, but it likely involves proteolytic cleavage of α- and γ-ENaC subunits [9,16]. Studies in *Xenopus *oocytes [13,14,17] or transfected mammalian cells [18] have demonstrated that trypsin-like serine proteases increase Na^+ ^transport by activating a population of near-silent channels rather than by promoting plasma membrane insertion of new channels. In mammals, the channel-activating proteases (CAP1,-2 and 3) are coexpressed with ENaC in epithelial tissues transporting Na^+ ^like renal collecting duct, lung, and colon [12,19,20]. Concerning the lung, we have recently shown that CAP1 is an important regulator of transepithelial alveolar Na^+ ^transport *in vitro *and *in vivo*, and of lung fluid homeostasis in the mouse [21,22]. Indeed, it was reported that Na^+ ^absorption across bronchial or nasal epithelial cells was regulated *in vitro *by endogenous aprotinin-sensitive serine protease(s) [15,23]. Prostasin, the human homolog of CAP1 expressed in proximal airways, was proposed as a likely candidate for this regulation [15,24].

Caldwell *et al *recently reported that ENaC activity and transepithelial Na^+ ^transport could be increased by apical treatment with human neutrophil elastase (hNE) in a human airway epithelial cell line [18]. However, it seems that this human airway epithelial cell line did not have any endogenous CAP activity inasmuch as treatment with aprotinin, an inhibitor of endogenous CAPs, did not modify transepithelial Na^+ ^transport. Whether hNE can also activate ENaC and Na^+ ^reabsorption in primary bronchial cells known to endogenously express CAPs is currently unknown. This is an important point inasmuch as hNE can be found at high concentration in airway surface liquid from CF patients, due to neutrophil activation. If hNE does activate ENaC and transepithelial Na^+ ^transport in CF airways, the use of hNE inhibitors could have a therapeutic interest for treatment of CF lung disease.

Our working hypotheses were (i) that hNE would stimulate ENaC and transepithelial Na^+ ^transport in primary human airway epithelial cells, and (ii) that EPI-hNE4, a specific and potent inhibitor of hNE [22], could block this stimulation. The objectives of the study were therefore to test the effects of hNE and EPI-hNE4 on ENaC activity and transepithelial Na^+ ^transport *in vitro *in primary cultures of human nasal epithelial cells from control and CF patients.

## Experimental Procedures

### Primary cultures of human nasal epithelial cells (HNEC)

Nasal polyps (NP) were obtained from non CF (n = 9) or CF (ΔF508/ΔF508, n = 4) patients requiring surgery for their nasal polyposis as previously described [25]. The diagnosis of nasal polyposis was established on the basis of clinical history, endoscopic findings and computed tomography results. This protocol was approved by the Institutional Review Board and ethics committee of our institution (CPP, Hôpital Henri Mondor), and informed consent was obtained from all patients. NP samples were immediately placed in DMEM/F12 supplemented with antibiotics (100 U/ml of penicillin, 100 mg/ml of streptomycin, 2.5 μg/ml of amphotericin B and 100 mg/ml of gentamicin) and transported to the laboratory for cell isolation. Briefly, NP samples were rinsed in phosphate-buffered saline (PBS) with dithiothreitol (5 nM) and antibiotics (100 U/ml of penicillin, 100 mg/ml of streptomycin, 2.5 μg/mL of amphotericin B and 100 mg/ml of gentamicin) and then placed overnight at 4°C in a PBS-antibiotics solution containing 0.1% pronase. The samples were incubated in DMEM/F12 with 5% fetal calf serum (FCS) before centrifugation (1,500 rpm, 7 minutes). Cell pellets were then suspended in 0.25% trypsin-ethylenediamine tetra-acetic acid (EDTA) solution for 3 minutes and incubated in DMEM/F12-antibiotics with 10% FCS. Finally, HNEC were plated on permeable polycarbonate supports (Snapwell^®^, Costar, Cambridge, USA) (1 × 10^6^cells/cm^2^) for short-circuit measurements. All inserts had a diameter of 12-mm and were coated with type IV collagen. HNEC were incubated at 37°C in 5% CO_2_. For the first 24 hours, HNEC were incubated with 1 ml of DMEM/F12-antibiotics with 2% Ultroser G outside the insert and DMEM/F12-antibiotics with 10% FCS inside the insert. After 24 hours, medium was removed inside the inserts in order to place the cells at an air-liquid interface, and medium outside the inserts was then changed daily. Transepithelial resistance and transepithelial potential difference were measured every three days using a microvoltmeter (World Precision Instruments, Astonbury, UK). Experiments were performed 2-3 weeks after isolation.

### Electrophysiological studies

Measurements of short-circuit current (*I*_sc_), transepithelial potential difference, and transepithelial resistance were performed in Snapwell inserts mounted in vertical diffusion chambers and bathed with Ringer solution (pH 7.4) continuously bubbled with 5% CO_2_-95% air at 37°C. The apical and basolateral chambers were filled with (in mM): 137 NaCl, 5.6 KCl, 1.9 CaCl_2_, 1.2 MgCl_2_, 5.9 CH_3_COONa, 1.3 NaH_2_PO_4_, 10 HEPES and 10 glucose. PD was short-circuited to 0 mV with a voltage clamp (World Precision Instruments, Astonbury, UK) connected to the apical and basolateral chambers *via *Ag-AgCl electrodes and agar bridges in order to measure *I*_sc _by Ohm's law. *I*sc was allowed to stabilize, before adding the drugs.

### Treatment of HNEC cultures

Human neutrophil elastase (Serva Electrophoresis; final concentration: 10 or 33 μg/ml, equivalent to 0.2 and 0.66 U/ml, respectively), EPI-hNE4 (developed by Dyax Corp., Cambridge, MA; final concentration: 10 or 33 μg/ml), trypsin (Sigma; final concentration: 100 μg/ml, equivalent to 1,000 BAEE units/ml) or vehicle were added after establishing a stable *I*_sc _into the apical compartment and *I*_sc _was monitored for 30 to 60 min before apical addition of 10 μM amiloride, a specific inhibitor of ENaC. Amiloride-sensitive *I*_sc _was determined as the difference in current with and without amiloride (10 μM). In the second part of the study, the serine protease inhibitor aprotinin (Sigma; final concentration: 50 μg/ml, equivalent to 0.25 Trypsin Inhibitor Unit (TIU)/ml) was added into the apical compartment and *I*_sc _was monitored for 75 to 90 min before apical addition of hNE alone (final concentration: 33 μg/ml), or of EPI-hNE4 (final concentration: 33 μg/ml) followed by hNE (final concentration: 33 μg/ml).

### Statistical analysis

Data are expressed as% of baseline value (before addition of drug) or as changes in *I*_sc _(Δ*I*_sc _, representing the difference between the value of *I*_sc _at the end of exposure to drug or vehicle and the baseline *I*_sc _at the moment of drug or vehicle addition), and are presented as means ± SE of 4-10 filters per condition. Statistics were performed on Δ*I*_sc _values. Treatment groups were compared by one-way variance analyses and, when allowed by the F value, results were compared by the modified least significant difference (Statview Software). P < 0.05 was considered significant.

## Results

### Treatment of control and CF HNEC with hNE

HNEC cultures were derived from nine non-CF and four CF (homozygous ΔF508/ΔF508) subjects. Thirty-five individual normal HNEC and nineteen CF HNEC filters displayed high transepithelial resistance and stable *I*_sc _values, and could be used in this study. Electrophysiological properties of cultured HNEC from non CF and CF patients are presented in Table 1.

**Table 1:** Electrophysiological properties of cultured HNEC from normal and CF patients.

	Control HNEC	CF HNEC
PD (mV)	36.8 ± 2.18	57 ± 2.23 ***

*R*_te _(Ω.cm^2^)	969 ± 67.7	953 ± 48.1

*I*_sc _(μA/cm^2^)	41.8 ± 3.42	60.7 ± 3.19 **

Human nasal epithelial cells (HNEC) from control (non CF) and CF patients were grown for 14 to 21 days on semi-permeable transwell filters until transepithelial resistance developed. Transwell filters were mounted in Ussing chamber for measurement of voltage (PD) and short-circuit current (*I*_sc_). Transepithelial resistance (*R*_te_) was calculated with Ohm's law from *I*_sc _and PD. Results represent means ± SE of 25 individual filters from 9 separate cultures of non CF HNEC, and of 9 individual filters from 4 separate cultures of CF HNEC.

**, ***: significantly different from corresponding value in control HNEC group (P < 0.01 and P < 0.001, respectively).

**Figure 1:**
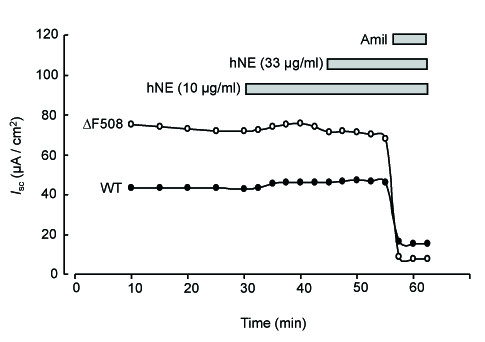
**Representative traces of short-circuit current measurements showing the effect of hNE in control and CF HNEC**. HNEC from control (WT) or CF patients (ΔF508) grown on Snapwell filters and mounted in Ussing chamber were exposed apically to hNE (final concentration: 10 and 33 μg/ml) for 30 minutes before amiloride (final concentration: 10 μM) was added.

**Figure 2:**
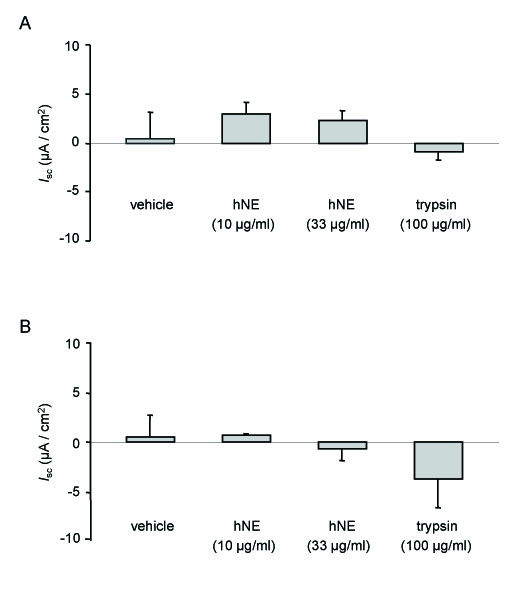
**Effect of hNE and trypsin on transepithelial Na^+ ^transport across control and CF HNEC**. HNEC from control (panel A) or CF (ΔF508) patients (panel B) grown on Snapwell filters and mounted in Ussing chamber were exposed apically to hNE, trypsin or vehicle. Δ*I*_sc _was calculated as the difference between the value of short-circuit current (*I*_sc_) at the end of exposure to drug or vehicle and the baseline *I*_sc _at the moment of drug or vehicle addition. Results are expressed in μA/cm^2 ^and represent means ± SE of five to seven filters for each condition: vehicle, hNE (10 μg/ml), hNE (33 μg/ml), and trypsin (100 μg/ml).

We first tested the effect of hNE on transepithelial Na^+ ^transport in control and CF HNEC monolayers. As shown in Figure 1, increasing concentrations of hNE (final concentration in the apical bath: 10 and 33 μg/ml) did not induce any noticeable change in *I*_sc _value in control HNEC. Indeed, Δ*I*_sc _(representing the difference between the value of *I*_sc _at the end of exposure to drug or vehicle and the baseline) was not significantly different in cells treated with hNE as compared with vehicle (Figure 2A) (n = 4-6). Treatment with excess trypsin (final concentration: 100 μg/ml), a serine protease known to activate ENaC and Na^+ ^transport in lung epithelial cells, also did not modify *I*_sc _value in control HNEC (Figure 2A). Similar results were obtained in CF HNEC since neither hNE (final concentration in the apical bath: 10 and 33 μg/ml) nor trypsin (final concentration: 100 μg/ml) did significantly modify Δ*I*_sc _as compared with vehicle (n = 3-4) (Figure 1 and 2B). These data show that, in cultured HNEC, ENaC-mediated transepithelial Na^+ ^transport could not be stimulated by treatment with exogenous serine proteases such as hNE and trypsin.

### Treatment of control and CF HNEC with EPI-hNE4

We next studied the effect of the hNE inhibitor EPI-hNE4 on control and CF HNEC to test whether this compound was able to modify transepithelial Na^+ ^transport. Treatment with a high concentration of EPI-hNE4 (final concentration: 100 μg/ml) did not significantly modify *I*_sc _in control HNEC (n = 5) or CF HNEC (n = 4), as compared with vehicle (Table 2).

**Table 2:** Effect of the hNE inhibitor EPI-hNE4 on Δ*I*_sc _in HNEC from control and CF patients.

	Control HNEC		CF HNEC	
	
	Vehicle	EPI-hNE4 (100 μg/ml)	Vehicle	EPI-hNE4 (100 μg/ml)
Δ*I*_sc _(μA/cm^2^)	0.5 ± 2.60	-0.9 ± 2.91	0.2 ± 2.15	-0.3 ± 2.52

Human nasal epithelial cells (HNEC) from control (non CF) and CF patients grown for 14 to 21 days on semi-permeable transwell filters were mounted in Ussing chamber and treated apically with either the hNE inhibitor EPI-hNE4 (final concentration 100 μg/ml) or vehicle. Δ*I*_sc _represents the difference between final *I*_sc _value at the end of experiment and basal *I*_sc _value before apical addition of vehicle or EPI-hNE4 in control and CF HNEC. Results are expressed as means ± SE (n = 4-5 individual filters for each condition).

### Effect of hNE and EPI-hNE4 treatment in control and CF HNEC preincubated with aprotinin

**Figure 3:**
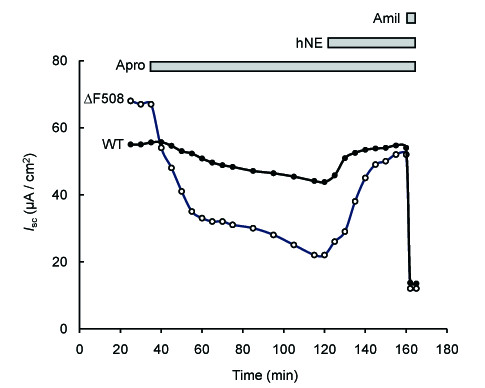
**Representative traces of short-circuit current measurements showing the effect of hNE in control and CF HNEC incubated with aprotinin**. HNEC from control (WT) and CF (ΔF508) patients grown on Snapwell filters were mounted in Ussing chamber and incubated apically with aprotinin (final concentration: 50 μg/ml) for 90 min before hNE (final concentration: 33 μg/ml) was added.

**Figure 4:**
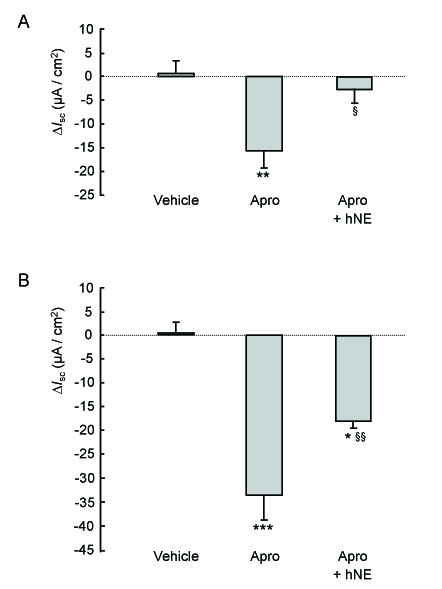
**Effect of hNE on transepithelial Na^+ ^transport across control and CF HNEC treated with aprotinin**. HNEC from control (panel A) and CF (ΔF508) (panel B) patients grown on Snapwell filters and mounted in Ussing chamber were exposed apically to vehicle, aprotinin (50 μg/ml), or aprotinin (for 90 min) followed by hNE (33 μg/ml). Δ*I*_sc _was calculated as the difference between the value of short-circuit current (*I*_sc_) at the end of exposure to drug or vehicle and the baseline *I*_sc _at the moment of drug or vehicle addition. Results are expressed in μA/cm^2 ^and represent means ± SE of four to ten filters for each condition. **, ***: significantly different from vehicle (P < 0.01 and P < 0.001, respectively); §, §§: significantly different from aprotinin alone (P < 0.05 and P < 0.01, respectively).

Taken together, the results indicate that neither hNE nor its inhibitor EPI-hNE4 is able to modify ENaC-mediated Na^+ ^transport in HNEC. We hypothesized that this lack of effect of hNE was due to the expression in HNEC of endogenous serine proteases known to activate ENaC in human airway epithelial cells, such as CAPs. To test this hypothesis, cells were pre-incubated with aprotinin (final concentration: 50 μg/ml in the apical bath), a Kunitz-type serine protease inhibitor, before hNE (with or without EPI-hNE4) was added. Aprotinin induced a 27.6 ± 3.47% decrease in control HNEC total *I*_sc _that was completely achieved within 90 min (Figure 3, 4 and Table 3). This decrease was rapidly and completely reversed by apical addition of hNE (final concentration 33 μg/ml). The stimulatory effect of hNE on *I*_sc _was fully prevented when cells were treated with EPI-hNE4 (final concentration 33 μg/ml) 5 minutes before hNE addition (Δ*I*_sc_: -15.7 ± 3.56 vs -14.2 ± 4.70 μA/cm^2 ^for aprotinin alone and aprotinin followed by EPI-hNE4 + hNE, respectively; NS).

**Table 3:** Comparison of the effects of aprotinin and hNE on *I*_sc _in control and CF HNEC.

	**Change in *I***_**sc **_**μA/cm**^**2 **^**(% baseline *I*_sc_)**	
	
	Aprotinin	hNE
Control HNEC	-15.7 ± 3.56 (-27.6 ± 3.47%)	9.5 ± 1.80 (18.5 ± 3.33%)

CF HNEC	-33.9 ± 5.20 * (-54 ± 8.18%) **	20.4 ± 4.47 (32.1 ± 6.7%)

Human nasal epithelial cells (HNEC) from control and CF patients were grown for 14 to 21 days on semi-permeable transwell filters until transepithelial resistance developed. Transwell filters were mounted in Ussing chamber for *I*_sc _measurements, and exposed apically to vehicle, aprotinin (50 μg/ml), or aprotinin (for 90 min) followed by hNE (33 μg/ml). The change in *I*_*sc *_induced by aprotinin represents the difference between the value of *I*_*sc *_at the end of aprotinin exposure and baseline *I*_*sc *_at the moment of aprotinin addition. The change in *I*_*sc *_induced by hNE represents the difference between peak *I*_*sc *_value following hNE addition and *I*_*sc *_value at the moment of hNE addition. Results are expressed in μA/cm^2 ^and in percentage of baseline *I*_*sc*_, and represent means ± SE of 4 to 10 filters for each condition. *, **: significantly different from corresponding value in control HNEC group (P < 0.05 and P < 0.01).

In CF HNEC, aprotinin decreased total *I*_sc _by 54 ± 8.18% in CF HNEC (Figure 3, 4 and Table 3). The aprotinin-induced inhibition of *I*_sc _was significantly greater in CF HNEC than in control HNEC (Table 3). Apical addition of hNE significantly increased *I*_sc _in aprotinin-treated CF HNEC. HNE-induced stimulation of *I*_sc _tended to be higher in CF HNEC than in control HNEC, although the difference was not significant (p = 0.06) (Table 3). However, hNE addition did not completely restore *I*_sc _to the baseline value in CF HNEC (Figure 3 and 4). The stimulatory effect of hNE in aprotinin-treated CF HNEC was completely blocked by preincubation with EPI-hNE4 (n = 2).

## Discussion

This study was designed to test the effect of hNE and its specific inhibitor EPI-hNE4 on transepithelial Na^+ ^transport across cultured normal and CF HNEC. Our results showed that neither hNE nor trypsin treatment did modify *I*_sc _in normal and CF HNEC, suggesting that ENaC at cell surface was already fully activated by endogenous serine proteases such as epithelial CAPs. Indeed, inhibition of endogenous CAPs with aprotinin induced a sustained decrease in *I*_sc _in both normal and CF HNEC, supporting this hypothesis. Interestingly, apical treatment with exogenous hNE completely and rapidly reversed the aprotinin-induced decrease in *I*_sc _normal and CF cells. EPI-hNE4 by itself did not modify *I*_sc _in normal or CF HNEC, indicating that this compound is a specific inhibitor of hNE and in this way could not inhibit endogenous CAPs. However, EPI-hNE4 completely abolished the stimulatory effect of hNE in cells pretreated with aprotinin in normal and CF patients. Taken together, our results suggest that in some conditions when endogenous CAPs are downregulated, hNE could stimulate ENaC-mediated Na^+ ^transport in both normal and CF HNEC, and that EPI-hNE4 could potently block this effect.

Mucus clearance is a major component of the lung innate defence mechanism. The efficiency of mucus clearance is partly dependent on the volume of airway surface liquid (ASL) on airway surfaces. The ASL is comprised of a periciliary liquid layer, which lubricates the cell surface, and a mucus layer, which traps airborne particules and pathogens. Cystic fibrosis airways exhibit Na^+ ^hyperabsorption and Cl^- ^hyposecretion, which leads to ASL volume depletion, mucus stasis and mucus plugging, which promote persistent bacterial infections [1]. Recent findings yielded novel insights into the role of ENaC hyperactivity in the *in vivo *pathogenesis of CF. Mall *et al *have demonstrated in a mouse model, that overexpression of the β-subunit of ENaC was sufficient to increase airway Na^+ ^absorption *in vivo *[6]. In this animal model, elevated airway Na^+ ^absorption caused airway surface liquid depletion, reduced mucus clearance, and deficient mucus clearance produced spontaneous lung disease sharing key features with CF [6,26]. Because ENaC hyperactivity in the airways is thought to play a key role in the pathogenesis of CF, decreasing ENaC-mediated Na^+ ^transport represents a therapeutic target to control ASL volume in CF airways.

It has been recently shown that ENaC channels expressed at the cell surface can be activated *in vitro *and *in vivo *by various trypsin-like serine proteases [10-14,17,27]. Membrane-bound Channel-Activating Proteases, which are co-expressed with ENaC in airway and alveolar epithelial cells [15,20-22,24] but also in other epithelial cells transporting Na^+ ^[12,14] have the ability to stimulate ENaC activity by increasing the channel opening probability, most likely through proteolytic cleavage of γ-ENaC subunit [9,16]. The effect of CAPs in lung epithelial cells is mimicked *in vitro *by trypsin, a serine protease which is normally not present in lung tissue [15,21,27]. Human neutrophil elastase is another serine protease present in CF airways at high concentrations due to the unrelenting infection and inflammation of the airways. Interestingly, Caldwell *et al *have recently reported that ENaC activity and transepithelial Na^+ ^transport could be increased by apical treatment with hNE in a human airway epithelial cell line [18]. The mechanism whereby hNE could activate ENaC function has been further analyzed in the *xenopus laevis *oocyte expression system. Harris *et al *have demonstrated that hNE could cleave the γ subunit of ENaC at cell surface [28]. It can be therefore hypothesized that ENaC activation by hNE *in vivo *could in some way contribute to ENaC hyperactivity encountered in CF airways. However, as the models previously used to study the effect of hNE, either airway epithelial cell lines or *xenopus laevis *oocytes, may be far from the *in vivo *conditions, we found it useful to study the effect of hNE on Na^+ ^transport across both normal and CF human primary epithelial cells.

Our experiments showed that apical addition of increasing concentrations of hNE did not significantly modify transepithelial Na^+ ^transport as assessed by I_sc _measurements in normal or CF HNEC. These results are in sharp contrast with those obtained by Caldwell et al in a human airway cell line [18]. Yet, they are not really surprising as previous studies have demonstrated that apical treatment with the exogenous serine protease trypsin had no effect on sodium current in human nasal epithelial cells and in rat alveolar epithelial cells [15,21], suggesting that in these primary cells, ENaC was fully activated by epithelium-derived serine proteases such as CAPs. It is important to note that the cell line used by Caldwell *et al *obviously did not show any endogenous CAP activity inasmuch as aprotinin (a non specific CAP inhibitor) incubation did not decrease Na^+ ^transport [18]. Therefore, we hypothesized that the lack of effect of hNE on *I*_sc _in our experiments was due to the fact that ENaC channels, once inserted in the plasma membrane, were already maximally activated by CAPs so that hNE could not further increase ENaC activity. Consistent with this hypothesis, we demonstrated that hNE was able to activate ENaC and transepithelial Na^+ ^transport in both normal and CF HNEC, but only when endogenous serine proteases such as CAPs were inhibited by aprotinin. Of note, we observed that inhibition of Na^+ ^transport by aprotinin was significantly larger in CF HNEC than in control HNEC. This finding, in line with what was previously reported by Myerburg et al [29], suggests that the activity of epithelial serine proteases, most likely CAPs, is increased in CF airways. We also noticed that, although hNE-stimulated *I*_sc _after aprotinin tended to be larger in CF than in control HNEC, hNE treatment failed to restore Na^+ ^transport at baseline value in CF cells, unlike in control cells. This suggests that in CF HNEC, hNE cannot fully substitute for aprotinin-sensitive epithelial serine proteases.

Another objective of the study was to test the effect of EPI-hNE4, a specific and potent inhibitor of hNE derived from the second Kunitz-type domain of inter-α-inhibitor protein [22,28], on ENaC and transepithelial Na^+ ^across primary HNEC. Our data show that under baseline conditions, EPI-hNE4 by itself did not modify *I*_sc _in normal or CF HNEC. This indicates that EPI-hNE4 does not inhibit CAP activity in these cells, which is not really surprising considering the fact that this compound is highly selective for hNE. However, EPI-hNE4 completely blocked the increase in *I*_sc _induced by hNE in cells first incubated with aprotinin.

Taken together, our results indicate that hNE is a potent activator of ENaC in primary nasal epithelial cells, but the physiological importance of this effect is questionable, inasmuch as ENaC seems to be constitutively maximally activated by epithelium-derived serine proteases such as CAPs, at least under physiological conditions. As far as we could see, EPI-hNE4 potently inhibited hNE, but failed to inhibit endogenous epithelium-derived CAPs, at least at the concentration used in this study. Yet, the present study does not rule out the possibility that, under pathological conditions such as airway inflammation encountered during CF, activation of ENaC by excess hNE released by neutrophils could be important. Interestingly, it has been reported that prostasin (CAP1) expression was markedly decreased in renal epithelial cells (M1 cell line) treated with TGFβ-1, a prototypic inflammatory cytokine [30]. Therefore, one can speculate that the expression and activity of endogenous CAPs might as well be reduced during airway inflammation, and that the stimulatory effect of hNE on ENaC could be unmasked. On this condition, the use of EPI-hNE4 could be of interest to reduce ENaC hyperactivity in CF airways. In order to elucidate the role of hNE on transepithelial Na^+ ^transport under inflammatory conditions, we intend to expose HNEC to prototypic inflammatory cytokines such as TGFβ-1 or IL1-β, which are known to decrease ENaC activity in these cells [25,30,31], and to study the effect of hNE.

## Competing interests

The authors declare that they have no competing interests.

## Authors' contributions

VPE carried out primary cultures from human nasal epithelial cells, short-circuit measurements, analysis and interpretation of data and participated to draft the manuscript.

CC participated in the study design and coordination.

GV participated in the study design and provided EPI-hNE4.

AC has been involved in revising this study before its submission.

EE has been involved in revising this study before its submission

CP conceived and designed the study and participated in its coordination, statistical analysis and helped to draft the manuscript.

All authors read and approved the final manuscript.
